# Morbid Itching of the Scrotum

**Published:** 1830-04-01

**Authors:** 


### XXIV.

### Morbid Itching of the Scrotum.

Many works are not adapted for a circumstantial analysis in the Review department of this Journal, but will yet furnish so much information on particular topics as not to deserve being passed altogether in silence. Of such a nature is a book lately published by Dr. Titley.* Though one which we would recommend strongly to the notice of our readers, and especially to the junior members of the profession, it is too elementary for a review or an analysis. We shall accordingly content ourselves with selecting some passages and noticing observations on points not generally understood, and commence with the section on itching of the scrotum.

"The skin of the scrotum is not unfrequently affected with a most troublesome itching. Sometimes this is occasioned by ascarides in the rectum; sometimes by morpiones, or by friction from violent exercise in hot weather; and occasionally from a morbid state of the skin, or superficial glands of the part. In the latter case the scrotum assumes a brown colour, and becomes thickened, scaly, and wrinkled; the itching extends to the skin covering the penis, more especially along the

* A Practical Treatise on the Diseases of the Genitals of the Male. 8vo. London, 1829.

course of the urethra, and the patient has little respite day or night.

"When the itching arises from ascarides in the rectum, the remedies suitable for the removal of these should be employed. When it it caused by morpiones, these may be destroyed by strong mercurial ointment rubbed on the part; by washing it with a solution of the oxymuriate of mercury, or by bathing the part freely with a decoction of stavesacre seed. Equal parts of calomel and starch, used as a powder, form an excellent application. If the itching has arisen simply from the excoriation consequent upon friction, a solution of the acetate of lead, or sulphate of zinc, are the most effectual remedies.

"When the disease is dependent on some morbid condition of skin, it is very difficult of cure; it commonly occurs in old men, and renders life truly miserable. Sulphur applied to the part, and sulphureous waters taken internally, are sometimes useful; but I must confess that I have often witnessed their unsuccessful employment. In some cases, I have used with much benefit a lotion composed of the emulsion of bitter almonds with oxymuriate of mercury, at the same time giving Plummer's pill at night, with a powder containing a scruple of soda and a drachm of sulphur, three times a day. As a general rule, greasy applications aggravate the complaint, but in two cases I have used, with much benefit, an unguent composed of one drachm of powder of opium, with an equal quantity of carbonate of soda, to an ounce of lard."

